# Evaluating Secukinumab as Treatment for Axial Spondyloarthritis and Psoriatic Arthritis in Patients with Comorbidities: Multicenter Real-Life Experience

**DOI:** 10.3390/jcm14155181

**Published:** 2025-07-22

**Authors:** Tuğba Ocak, Burcu Yağız, Belkıs Nihan Coşkun, Gamze Akkuzu, Ayşe Nur Bayındır Akbaş, Özlem Kudaş, Elif İnanç, Özge Yoğurtçu, Fatma Başıbüyük, Sezgin Zontul, Fatih Albayrak, Zeynel Abidin Akar, Saliha Sunkak, Selime Ermurat, Dilek Tezcan, Adem Küçük, Servet Yolbaş, İsmail Sarı, Murat Yiğit, Servet Akar, Bünyamin Kısacık, Cemal Bes, Ediz Dalkılıç, Yavuz Pehlivan

**Affiliations:** 1 Department of Rheumatology, Bursa City Hospital, 16250 Bursa, Turkey; 2 Division of Rheumatology, Department of Internal Medicine, Faculty of Medicine, Uludağ University, 16059 Bursa, Turkey; burcuyilmaz_84@hotmail.com (B.Y.); belkisnihanseniz@hotmail.com (B.N.C.); edizinci@hotmail.com (E.D.); drypehlivan@gmail.com (Y.P.); 3 Department of Rheumatology, Başakşehir Çam and Sakura City Hospital, Health Sciences University, 34480 İstanbul, Turkey; gamzeakkuzu89@hotmail.com (G.A.); cemalbes@hotmail.com (C.B.); 4 Division of Rheumatology, Department of Internal Medicine, Faculty of Medicine, Pamukkale University, 20160 Denizli, Turkey; draysenurbayindir@gmail.com (A.N.B.A.); drmuratygt@gmail.com (M.Y.); 5 Division of Rheumatology, Istanbul Physical Therapy Rehabilitation Training and Research Hospital, 34180 Istanbul, Turkey; ozlemkudas@gmail.com; 6 Division of Rheumatology, Department of Internal Medicine, Faculty of Medicine, İnönü University, 44280 Malatya, Turkey; elif.temelli@hotmail.com (E.İ.); servetyolbas@yahoo.com.tr (S.Y.); 7 Division of Rheumatology, Department of Internal Medicine, Faculty of Medicine, İzmir Katip Çelebi University, 35620 İzmir, Turkey; hatipogluozge91@gmail.com (Ö.Y.); servet.akar@gmail.com (S.A.); 8 Division of Rheumatology, Department of Internal Medicine, Faculty of Medicine, Dokuz Eylül University, 35390 İzmir, Turkey; mdfatmabasibuyuk@gmail.com (F.B.); ismailsari35@gmail.com (İ.S.); 9 Division of Rheumatology, Department of Physical Therapy and Rehabilitation, Faculty of Medicine, Inönü University, 44280 Malatya, Turkey; sezginzontul@hotmail.com; 10 Department of Internal Medicine, Division of Rheumatology, Gaziantep Medical Faculty Hospital, 27310 Gaziantep, Turkey; drfalbayrak@yahoo.com; 11 Department of Physical Therapy and Rehabilitation, Division of Rheumatology, Dicle University Hospital, 21280 Diyarbakır, Turkey; zeynelabidin_akar@yahoo.com; 12 Department of Rheumatology, Kayseri City Education and Research Hospital, 38080 Kayseri, Turkey; sadogan.84@gmail.com; 13 Department of Rheumatology, Bursa Yüksek Ihtisas Training and Research Hospital, 16140 Bursa, Turkey; selimeermurat@hotmail.com; 14 Division of Rheumatology, Department of Internal Medicine, Gülhane Faculty of Medicine, University of Health Sciences, 06018 Ankara, Turkey; dr_dilekturan@hotmail.com; 15 Department of Internal Medicine, Division of Rheumatology, Faculty of Medicine, Necmettin Erbakan University, 42090 Konya, Turkey; drademk@gmail.com; 16 Department of Rheumatology, Sanko University Medical Faculty Hospital, 16049 Gaziantep, Turkey; bunyamin.kisacik@yahoo.com

**Keywords:** congestive heart failure, multiple sclerosis, secukinumab, tuberculosis

## Abstract

**Background**: Secukinumab is a fully human monoclonal antibody that targets interleukin (IL)-17A and is used to treat axial spondyloarthritis (axSpA) and psoriatic arthritis (PsA). Treating axSpA and PsA can be challenging in patients with comorbidities. In this multicenter retrospective study, we aimed to evaluate the efficacy and safety of secukinumab treatment in patients with axSpA and PsA who had a history of tuberculosis, multiple sclerosis (MS), or congestive heart failure (CHF). **Methods**: The study included 44 patients with a diagnosis of axSpA and PsA and a history of tuberculosis, MS, or CHF who received secukinumab treatment at 13 centers in our country. Erythrocyte sedimentation rate, C-reactive protein (CRP), Bath Ankylosing Spondylitis Disease Activity Index, Ankylosing Spondylitis Disease Activity Score CRP, visual analog scale, and Disease Activity Score-28 CRP markers at months 0, 3, and 12 of secukinumab treatment were analyzed. Alongside this, tuberculosis, MS, and CHF were evaluated at follow-up using clinical assessments and imaging methods such as chest radiographs, brain magnetic resonance, and echocardiography. **Results**: A statistically significant improvement in inflammatory markers and disease activity scores was observed in patients treated with secukinumab. There was no reactivation in patients with a history of tuberculosis. In most MS patients, the disease was stable, while clinical and radiological improvement was observed in one patient. No worsening of CHF stage was observed in patients with a history of CHF. **Conclusions**: With regular clinical monitoring, secukinumab may be an effective and safe treatment option for axSpA and PsA patients with a history of tuberculosis, MS, or CHF.

## 1. Introduction

Axial spondyloarthritis (axSpA) and psoriatic arthritis (PsA) are chronic systemic inflammatory diseases within the spondyloarthritis (SpA) spectrum. Both may lead to a deterioration in quality of life and structural damage if left untreated [1,2]. Non-steroidal anti-inflammatory drugs (NSAIDs) and conventional synthetic disease-modifying antirheumatic drugs (csDMARDs) such as sulfasalazine and methotrexate have long been used to treat AxSpA and PsA. However, for patients who do not respond to these treatments, biologic agents, particularly tumor necrosis factor (TNF) inhibitors, have provided a significant advance in treatment [3,4].

Some patients do not respond to TNF inhibitors or cannot continue this treatment due to side effects. This has led to the development of alternative treatment strategies. In recent years, the interleukin (IL)-17 signaling pathway has been shown to play a crucial role in the pathogenesis of SpA. Secukinumab, a human monoclonal antibody that selectively inhibits IL-17A, has also been developed. In patients with PsA, IL-17 and IL-23 targeting agents are preferred when skin manifestations are prominent [5], and secukinumab has been shown to improve clinical and radiological outcomes in both axSpA and PsA patients [6,7].

Although the efficacy and safety of secukinumab have been demonstrated in randomized controlled trials, patients with a history of comorbidities such as tuberculosis, multiple sclerosis (MS), or congestive heart failure (CHF) are generally not included in these trials [6,7]. This limits the availability of real-world data on the efficacy and safety of secukinumab in high-risk patient groups.

The risk of reactivation of tuberculosis by TNF inhibitors is a generally recognized consequence of this type of treatment and may limit the use of these drugs, particularly in countries where tuberculosis is endemic or in people with a history of tuberculosis [8,9]. The risk of tuberculosis reactivation is thought to be lower with IL-17 inhibitors due to their different mechanisms of action, but real-world data on this are scarce [10,11]. Similarly, TNF inhibitors have been reported to exacerbate demyelinating diseases, and the use of TNF inhibitors is contraindicated in patients with a history of MS [12]. The IL-17 family of cytokines and Th17 cells have been shown to play important roles in the pathogenesis of MS, and IL-17A and IL-17F have been shown to increase the pro-inflammatory response in the endothelial and glial cells in the central nervous system [13]. These data suggest that the use of secukinumab may be a treatment option for axSpA and PsA patients with a history of MS [13,14,15]. Furthermore, it has been shown that CHF may develop or be exacerbated with TNF inhibitors [16,17,18]. In a randomized controlled trial with high-dose infliximab, a worsening of the clinical condition was observed in patients with stage 3 and 4 CHF [19]. Based on these results, TNF inhibitors are not advised for patients with CHF [20]. However, secukinumab is conditionally recommended in these patients [20], as a limited number of studies have assessed the use of secukinumab in the context of CHF [21,22,23].

Comorbidities such as tuberculosis, MS, or CHF complicate the treatment management of patients with axSpA and PsA. The existing studies on these patient groups are generally case series or studies with small sample sizes [21,22,23,24,25,26,27,28]. In our multicenter, real-world study, we aimed to evaluate the efficacy and safety of secukinumab treatment in axSpA and PsA patients with a history of tuberculosis, MS, or CHF.

## 2. Materials and Methods

### 2.1. Study Design and Patient Population

**Figure 1:**
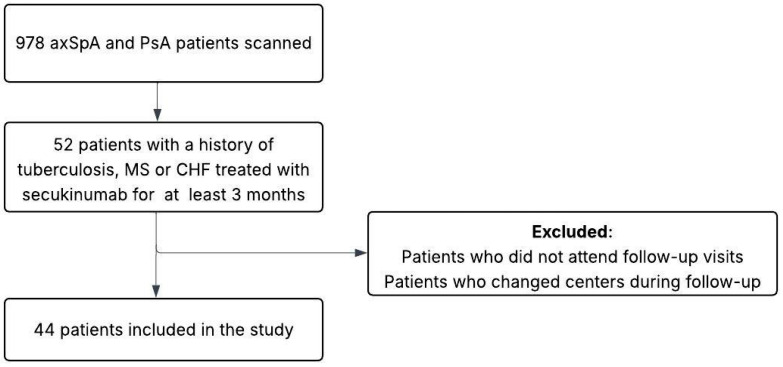
A diagram of the study design. AxSpA = Axial spondyloarthritis; PsA = Psoriatic arthritis; MS = Multiple sclerosis; CHF = Congestive heart failure.

Secukinumab was approved for the treatment of axSpA and PsA in Turkey in May 2018 [29]. Our multicenter study included 13 centers in Turkey. In these centers, 978 patients aged 18 years and older with a diagnosis of axSpA or PsA according to the classification criteria of the Assessment of SpondyloArthritis International Society (ASAS) criteria [30] and the classification criteria for psoriatic arthritis (CASPAR) [31], who were treated with secukinumab between May 2018 and November 2024, were retrospectively reviewed. There were 52 patients who had been diagnosed with tuberculosis, MS, or CHF before starting treatment with secukinumab and who had been treated with the drug for at least three months. Patients attended regular follow-up visits every 3 months. Patients who did not attend the follow-up visits or went to another center during the follow-up visits were excluded from the study. Forty-four patients were ultimately included in the study. A study flow chart is presented in Figure 1. Secukinumab was administered subcutaneously to patients with axSpA at a dosage of 150 mg (once a week for four weeks, then once every four weeks) and to patients with PsA at a dosage of 300 mg (once a week for four weeks, then once every four weeks). Patients who had not received biologic therapy before treatment with secukinumab were defined as bionaive, and patients who had received biologic therapy before treatment with secukinumab were defined as bioexperienced. In bioexperienced patients, secukinumab treatment was initiated due to active SpA, adverse events related to prior treatment, or the availability of secukinumab.

All patients with a history of active tuberculosis had received tuberculosis treatment at the appropriate dose and duration before treatment with secukinumab. All patients were examined every 3 months for clinical symptoms and every 6 months using chest radiographs to assess the reactivation of tuberculosis. In MS, disease activity was as-sessed by the progression of clinical symptoms or brain magnetic resonance (MR) imaging of the lesions before and after treatment with secukinumab. Regression of both clinical symptoms and MR findings was considered a response to treatment. Unchanged clinical symptoms or MR findings were considered stable disease; worsening of clinical symptoms or an increase in MR lesions was considered progression [26]. CHF was categorized according to the New York Heart Association (NYHA) [32]. CHF was evaluated based on the left ventricular ejection fraction (LVEF) via echocardiography. Heart failure with reduced ejection fraction (HFrEF) is defined as an LVEF < 40%. Heart failure with mildly reduced ejection fraction (HFmrEF) is defined as an LVEF between 41% and 49% [33]. Patients with CHF were assessed for clinical symptoms every 3 months, and the LEVF was assessed via echocardiography before and after treatment with secukinumab.

### 2.2. Data Collection

Gender, age, smoking and alcohol usage, comorbidities, human leukocyte antigen (HLA) B27 positivity, the presence of enthesitis, dactylitis, and uveitis, the types of bi-ological therapies before secukinumab treatment, the reasons for discontinuation of previous biological therapies, and the duration of secukinumab treatment were recorded. For the assessment of response to secukinumab treatment, a subgroup analysis was performed for patients treated with secukinumab for at least 12 months. Erythrocyte sedimentation rate (ESR), C-reactive protein (CRP) levels, and pain based on a 10 cm visual analog scale (VAS) [34] were evaluated at 0 months, 3 months, and 12 months in patients treated with secukinumab for at least 12 months. Bath Ankylosing Spondylitis Disease Activity Index (BASDAI) [35] and Ankylosing Spondylitis Disease Activity Score (ASDAS) CRP [36] results were analyzed at 0 months, 3 months, and 12 months in patients with axSpA and PsA with axial involvement who were treated with secukinumab for at least 12 months. Disease Activity Score-28 (DAS 28) CRP [37] was assessed at 0 months, 3 months, and 12 months only in patients with PsA with peripheral involvement. Psoriasis Area and Severity Index (PASI) [38] scores were evaluated in PsA patients with active skin involvement. Patients with PASI scores between 10 and 20 were classified as having moderate-to-severe disease activity, while those with PASI scores >20 were considered to have severe disease activity. PASI 75 is defined as a 75% improvement in the PASI [39]; PASI 75 responses in these patients were evaluated at the last visit of secukinumab treatment.

Age at tuberculosis diagnosis, the time between tuberculosis diagnosis and initiation of secukinumab treatment, and tuberculosis reactivation status during follow-up were analyzed in patients with a history of tuberculosis. Age at MS diagnosis, time between MS diagnosis and initiation of secukinumab treatment, MS treatments before and with secukinumab, and MS disease status (response to treatment, stable disease, progressive disease) at the last secukinumab treatment visit were evaluated in patients with a history of MS. In patients with a history of CHF, the age at diagnosis of CHF, the time between the diagnosis of CHF and the start of treatment with secukinumab, and the stage of CHF before secukinumab and at the end of the follow-up period were analyzed.

### 2.3. Statistical Analysis

Statistical analysis was conducted using SPSS (Statistical Package for Social Sciences) version 26.0. Descriptive statistics were used for the sociodemographic, clinical, and laboratory parameters of the patients. The normality of variables was assessed using the Shapiro–Wilk and Kolmogorov–Smirnov tests. Quantitative data are expressed as mean ± standard deviation for normally distributed data and median (minimum, maximum) for non-normally distributed data. The Friedman test was used to evaluate treatment response and determine whether there was a relationship between the ESR, CRP, BASDAI, ASDAS CRP, and VAS levels at months 0, 3, and 12. Then, the Wilcoxon signed-rank test was used for the comparisons between visits at months 0 and 3, 0 and 12, and 3 and 12. Bonferroni correction was performed for the three separate paired group comparisons, and a new significant *p*-value < 0.0167 was accepted. The correlations between ESR and ASDAS CRP, BASDAI, and VAS at months 0, 3, and 12, as well as the correlations between CRP and ASDAS CRP, BASDAI, and VAS at the same time points, were assessed using Spearman correlation analysis.

## 3. Results

### 3.1. Characteristics of Patients with a History of Tuberculosis

A total of 19 patients had a history of tuberculosis before treatment with secukinumab. The characteristics of these patients at the initiation of secukinumab treatment are shown in Table 1. The mean age at diagnosis of SpA was 31.8 ± 8 years. AxSpA was diagnosed in 17 patients (89.5%), and PsA with axial involvement in 2 patients (10.5%). The mean age at diagnosis of tuberculosis was 36.4 ± 14.2 years. Of the patients diagnosed with tuberculosis, 17 had pulmonary tuberculosis and 2 had peritoneal tuberculosis. Active tuberculosis developed during TNF inhibitor therapy in 12 bioexperienced patients. TNF inhibitors were discontinued due to tuberculosis. After receiving adequate tuberculosis treatment in terms of both dosage and duration, secukinumab was initiated in these patients due to active SpA. Among the bioexperienced patients, one with a diagnosis of axSpA was started on etanercept 30.1 years after the diagnosis of tuberculosis due to active SpA. As the patient exhibited disease activity under etanercept, secukinumab was initiated following its approval in our country. The median interval between the diagnosis of tuberculosis and the start of secukinumab treatment was 4.9 (1–39.8) years. A comparison of inflammatory markers and disease activity at 0, 3, and 12 months in patients with a history of tuberculosis treated with secukinumab for at least 12 months is shown in Table 2. There was a statistically significant difference between ESR, CRP, BASDAI, ASDAS-CRP, and VAS scores at months 0, 3, and 12 (*p* < 0.001, *p* = 0.003, *p* < 0.001, *p* < 0.001, and *p* < 0.001, respectively). At month 0, no significant correlations were found between ESR and ASDAS CRP, BASDAI, or VAS (*p* = 0.325, *p* = 0.225, and *p* = 0.352, respectively). At month 3, no significant correlations were detected between ESR and ASDAS CRP, BASDAI, or VAS (*p* = 0.384, *p* = 0.216, and *p* = 0.453, respectively). At month 12, no significant correlations were found between ESR and ASDAS CRP, BASDAI, or VAS (*p* = 0.696, *p* = 0.288, and *p* = 0.877, respectively). At month 0, a moderate correlation was found between CRP and ASDAS CRP (r = 0.569, *p* = 0.011). No significant correlations were observed between CRP and BASDAI or between CRP and VAS at month 0 (*p* = 0.894 and *p* = 0.623, respectively). At month 3, a moderate correlation was found between CRP and ASDAS CRP (r = 0.596, *p* = 0.007). However, no significant correlations were found between CRP and BASDAI or VAS at month 3 (*p* = 0.198 and *p* = 0.144, respectively). At month 12, no significant correlations were observed between CRP and ASDAS CRP, BASDAI, or VAS (*p* = 0.429, *p* = 0.172, and *p* = 0.986, respectively). Among the patients diagnosed with PsA, one patient with a baseline PASI score in the moderate-to-severe range achieved a PASI 75 response at the last treatment visit. Treatment with secukinumab was discontinued in two patients after 3.2 and 6.2 months due to ineffectiveness. In patients with a history of tuberculosis, no reactivation of tuberculosis was observed during a median follow-up period of 19.4 (3.2–78.1) months of treatment with secukinumab. No significant side effects were observed during secukinumab treatment.

### 3.2. Characteristics of Patients with a History of MS

There were nine patients with a history of MS before treatment with secukinumab. The characteristics of these patients at the initiation of secukinumab are shown in Table 3. The mean age at diagnosis of SpA was 37.9 ± 9.6 years. Five patients (55.6%) were diagnosed with axSpA, 2 (22.2%) with PsA with axial involvement, and two (22.2%) were diagnosed with PsA with peripheral involvement. All patients had relapsing–remitting MS. Four of the bioexperienced patients were diagnosed with MS after the initiation of biologic therapy. In these patients, where biologic treatment was discontinued due to the MS diagnosis, secukinumab was initiated because of active SpA. Among the bioexperienced patients, two patients who were receiving ustekinumab had been diagnosed with MS before initiating biologic therapy, and no disease progression was observed under this treatment. In two bioexperienced patients who had been receiving ustekinumab, the reason for switching to secukinumab was active SpA. The mean interval between MS diagnosis and the start of secukinumab treatment was 3 ± 2.7 years. In terms of the MS treatments, seven patients were receiving intermittent steroid treatment, one patient was receiving azathioprine and intravenous immunoglobulin (IVIG), and one patient was receiving dimethyl fumarate before treatment with secukinumab. After treatment with secukinumab, four patients continued drug-free treatment, three patients continued intermittent steroid treatment, one patient continued IVIG treatment, and one patient continued dimethyl fumarate treatment. Table 4 shows the comparison of inflammatory markers and disease activity at months 0, 3, and 12 in patients with MS treated with secukinumab for at least 12 months. A statistically significant difference was observed between ESR, CRP, BASDAI, ASDAS CRP, and VAS scores at months 0, 3, and 12 (*p* < 0.001, *p* = 0.003, *p* < 0.002, *p* ≤ 0.001 and *p* < 0.001, respectively). In one of the patients with PsA with peripheral involvement, the DAS 28 CRP values were 5.8, 5.1, and 2.8 at months 0, 3, and 12, respectively. In the other patient, the DAS 28 CRP values were 5.2, 1.9, and 1.2 at months 0, 3, and 12, respectively. At month 0, no significant correlations were observed between ESR and ASDAS CRP, BASDAI, or VAS (*p* = 0.398, *p* = 0.756, and *p* = 0.208, respectively). Similarly, at month 3, ESR was not significantly correlated with ASDAS CRP, BASDAI, or VAS (*p* = 0.758, *p* = 0.908, and *p* = 0.677, respectively). At month 12, no significant correlations were found between ESR and ASDAS CRP, BASDAI, or VAS (*p* = 0.691, *p* = 0.574, and *p* = 0.533, respectively). At month 0, no significant correlations were observed between CRP and ASDAS CRP, BASDAI, or VAS (*p* = 1.000, *p* = 0.111, and *p* = 0.944, respectively). At month 3, CRP was not significantly correlated with ASDAS CRP, BASDAI, or VAS (*p* = 0.294, *p* = 0.058, and *p* = 1.000, respectively). At month 12, no significant correlations were found between CRP and ASDAS CRP, BASDAI, or VAS (*p* = 0.567, *p* = 0.483, and *p* = 0.569, respectively). Among the patients diagnosed with PsA, one patient with a baseline PASI score in the moderate-to-severe range achieved a PASI 75 response at the last visit of treatment. In one patient, treatment with secukinumab was discontinued after 15.7 months due to ineffectiveness. At the end of the median follow-up period of 67.8 (13.4–78.6) months, eight patients were stable and one patient responded to treatment in terms of MS improvement. No significant side effects were observed during secukinumab treatment.

### 3.3. Characteristics of Patients with a History of CHF

There were 16 patients with a history of CHF before treatment with secukinumab. The characteristics of these patients at the initiation of secukinumab are shown in Table 5. The median age at SpA diagnosis was 40 (33.4–65.9) years. Thirteen (81.2%) of the patients had axSpA, two (12.5%) had axial PsA, and one (6.3%) had peripheral PsA. Based on NYHA classification, nine patients had stage 2 CHF and seven had stage 3 CHF before secukinumab treatment. Based on LVEF classification, 11 patients were categorized as HFmrEF and 5 patients as HFrEF before secukinumab treatment. In three bioexperienced patients, biologic therapy was discontinued due to the presence of CHF during treatment. During follow-up, secukinumab was initiated because of the reactivation of SpA. In four other bioexperienced patients, biologic therapy was discontinued due to SpA remission. During follow-up, after the diagnosis of CHF, secukinumab was initiated due to the reactivation of SpA. The mean time between the diagnosis of CHF and the start of secukinumab treatment was 4.3 ± 3.2 years. Table 6 shows a comparison of inflammatory markers and disease activity at 0, 3, and 12 months in patients with a history of CHF who had been treated with secukinumab for at least 12 months. A statistically significant difference was found between patients’ ESR, CRP, BASDAI, ASDAS-CRP, and VAS scores at 0, 3, and 12 months (*p* ≤ 0.001, *p* < 0.001, *p* < 0.001, *p* < 0.001, and *p* < 0.001, respectively). At month 0, no significant correlations were found between ESR and ASDAS CRP, BASDAI, or VAS (*p* = 0.129, *p* = 0.362, and *p* = 0.670, respectively). At month 3, ESR was not significantly correlated with ASDAS CRP, BASDAI, or VAS (*p* = 0.462, *p* = 0.627, and *p* = 0.760, respectively). At month 12, no significant correlations were observed between ESR and ASDAS CRP, BASDAI, or VAS (*p* = 0.978, *p* = 0.277, and *p* = 0.637, respectively). At month 0, no significant correlations were observed between CRP and ASDAS CRP, BASDAI, or VAS (*p* = 0.052, *p* = 0.712, and *p* = 0.433, respectively). At month 3, a moderate correlation was found between CRP and ASDAS CRP (r = 0.738, *p* = 0.002). At month 3, no significant correlations were observed between CRP and BASDAI or VAS (*p* = 0.952 and *p* = 0.375, respectively). At month 12, CRP was not significantly correlated with ASDAS CRP, BASDAI, or VAS (*p* = 0.685, *p* = 0.310, and *p* = 0.688, respectively). In one patient with peripheral PsA, DAS 28 CRP was 5.2, 4.8, and 2.1 at 0, 3, and 12 months, respectively. Among the patients diagnosed with PsA, one patient with a baseline PASI score in the severe range achieved a PASI 75 response at the last visit of treatment. Secukinumab treatment was discontinued in one patient after 37.7 months due to ineffectiveness. At the end of the median follow-up period of 31.3 (14.3–69.7) months, relative to the start of secukinumab treatment, the CHF stage of the patients was stable according to the NYHA and LEVF classifications. No significant side effects were observed during secukinumab treatment.

## 4. Discussion

This multicenter, retrospective study is one of the few studies to investigate the efficacy and safety of using secukinumab to treat axSpA and PsA patients with a history of tuberculosis, MS, or CHF. In our study, secukinumab achieved a significant improvement in SpA-related clinical and laboratory parameters in these high-risk patient groups, and no activation of concomitant diseases was observed.

Since TNF is responsible for maintaining the granuloma structure, TNF inhibitors can lead to reactivation of latent mycobacterial infection [40,41]. Although IL-17 has been shown to play a role in neutrophil mobilization and mucosal immunity, it has also been found to be less effective than TNF in tuberculosis-specific defense responses such as granuloma formation [42]. Clinical and real-life data also show that secukinumab is safe to use in patients with latent tuberculosis infection. Elewski et al. conducted a pooled cohort study of 28 clinical trials examining psoriasis, PsA, and ankylosing spondylitis (AS) [43]. They found that of the 12,319 patients treated with secukinumab, 684 had latent tuberculosis infection, and no active cases of tuberculosis were reported for five years [43]. In the study by Liu et al., no reactivation of tuberculosis was observed in six secukinumab-treated axSpA patients with latent tuberculosis during a mean follow-up of 17.2 ± 10.4 months [11]. All 19 patients in our study who had tuberculosis prior to secukinumab treatment received an adequate duration and dose of antituberculosis treatment, and none of them experienced reactivation of tuberculosis during a median follow-up period of 19.4 months. Previous studies have investigated the reactivation of tuberculosis in patients with latent tuberculosis infection. However, our study is the first regarding the use of secukinumab in patients with a history of active tuberculosis. In addition, statistically significant decreases in ESR, CRP, BASDAI, ASDAS CRP and VAS scores were observed in this patient group, and only two patients discontinued treatment due to inefficacy. Based on these findings, secukinumab can be used in axSpA and PsA patients with a history of active tuberculosis if the patients are carefully monitored.

It is assumed that the immune system plays a role in the development of MS. Histopathological evaluation has shown that inflammatory T cells, B cells, and macrophages are active in MS plaques [44]. In animal models developed for MS and autoimmune encephalitis, Th17 cells, a novel IL-17-secreting CD4+ T cell subset, have been shown to play a role in disease pathogenesis. In active MS lesions, increased levels of IL-17 mRNA and IL-17-related products have been detected in the astrocytes, oligodendrocytes, and lymphocytes around blood vessels; moreover, the number of IL-17 + T cells is higher in active MS lesions than in inactive lesions [45]. In a randomized controlled trial of 73 patients, MR imaging was used to show that treatment with secukinumab reduced lesion activity in MS patients [46]. Studies on the treatment of MS with secukinumab are mainly case reports and case series [24,25,26,27,28]. Eksin et al. analyzed four case series of secukinumab-treated PsA and AS patients, as well as eight other cases described in the literature [27]. Of the 12 MS cases included in the study, a response to treatment was achieved in three patients during a median follow-up of 13.5 months, while the disease remained stable in nine patients [27]. In the review by Chaitidis et al. on MS or MS-like syndrome, 19 studies with 42 patients who received IL-17 inhibitors for any indication were analyzed. IL-17 inhibitors other than secukinumab were also included in this review [28]. Neurological relapse occurred in three patients receiving secukinumab during a median follow-up of 12.5 months [28]. It was suggested that the neurological relapses may have been due to the failure of secukinumab treatment to adequately stabilize the progression of MS, rather than an adverse effect of the treatment [28]. This review showed, with limited evidence, that IL-17 inhibition can be an effective and safe treatment for MS patients [28]. In our study, during the median follow-up period of 67.8 months, MS remained stable in eight patients, while one patient responded to treatment. Improvements in inflammatory markers and disease activity were also observed in the evaluation of axSpA and PsA. Our study, which has the largest number of patients in this particular group and the longest follow-up in the literature, shows that secukinumab may be a good treatment option for axSpA and PsA patients with a history of MS.

CHF is considered an inflammatory disease in which cytokines play a role. In animal models, elevated IL-17A levels have been linked to impaired cardiac function and fibrosis [47,48]. Baumhove and colleagues demonstrated that high serum IL-17 levels are associated with more severe CHF [49]. These findings suggest that agents such as secukinumab, which block IL-17A, may have a protective effect in CHF. However, practical experience in this area is limited. To our knowledge, there are a limited number of studies where patients with a history of CHF are treated with secukinumab [21,22,23]. One patient with non-alcoholic steatohepatitis, stage 2 CHF, and demyelinating disease was followed for 10 months and remained clinically stable under secukinumab treatment [21]. In another case, secukinumab treatment resulted in the resolution of psoriatic skin lesions and improvement in ejection fraction at the third month in a patient with PsA and acute CHF due to dilated cardiomyopathy [22]. In the study by Frustaci et al., increased IL-17A expression was observed in endomyocardial biopsies from five patients with dilated cardiomyopathy [23]. Two of these patients received secukinumab for six months, and complete recovery of LVEF was achieved with secukinumab treatment [23]. In our study, improvements in inflammatory markers and disease activity were observed in axSpA and PsA. Over a median follow-up period of 31.3 months, CHF remained stable in the patients. Moreover, in our study, which included the largest number of patients and the longest follow-up period to date, secukinumab was demonstrated to be an effective and safe treatment option for patients with axSpA and PsA who have a history of CHF.

Our study has some limitations. The follow-up time varied due to the heterogeneous nature of the patient population. Our study includes only the Turkish population, has a relatively small sample size and is retrospective in nature, which may affect the generalizability of our results. In addition, the absence of a comparative patient group treated with other biological agents makes it difficult to draw definitive conclusions about the relative efficacy and safety of this drug in the analyzed population. However, our study’s multicenter design and longer follow-up period compared to previous investigations are among its major strengths.

## 5. Conclusions

In this multicenter, retrospective study based on real-world data, the long-term efficacy and safety of secukinumab treatment were investigated in patients with axSpA and PsA and a history of tuberculosis, MS, or CHF. The results show that secukinumab significantly improves both inflammatory markers and disease activity scores in these high-risk patient groups, does not affect the clinical course of existing comorbidities, and does not cause serious side effects. The absence of reactivation in patients with a history of tuberculosis, the largely stable disease course in MS patients, and the lack of worsening in patients with CHF indicate that secukinumab can be considered for these patients, provided that regular clinical monitoring is adhered to.

## Figures and Tables

**Table 1:** Characteristics of patients with a history of tuberculosis at the start of secukinumab treatment (*n* = 19).

Diagnosis age of SpA, years	31.8 ± 8
Male sex, *n* (%)	17 (89.5)
SpA characteristics, *n* (%)	
HLA B27	9 (47.4)
AxSpA	17 (89.5)
PsA	2 (10.5)
Enthesitis	0 (0)
Dactylitis	1 (5.3)
Uveitis	0 (0)
Smoking, *n* (%)	
Current	10 (52.6)
Former	3 (15.8)
Never	6 (31.6)
Alcohol	
Current	4 (21.1)
Former	1 (5.3)
Never	14 (73.7)
Comorbidity, *n* (%)	
Hypertension	3 (15.8)
Diabetes mellitus	1 (5.3)
Dyslipidemia	2 (10.5)
Coronary artery disease	1 (5.3)
Chronic obstructive pulmonary disease	1 (5.3)
Hypothyroidism	1 (5.3)
Familial Mediterranean fever	1 (5.3)
Biological therapies, *n* (%)	
Bionaive	6 (31.6)
Bioexperienced *	13 (68.4)
Etanercept	4 (21.1)
Adalilumab	6 (31.6)
Certolizumab	2 (10.5)
Infliximab	6 (31.6)
Age at tuberculosis diagnosis, years	36.4 ± 14.2
Time between tuberculosis and treatment with secukinumab, years	4.9 (1–39.8)
Secukinumab follow up time, months	19.4 (3.2–78.1)

SpA = spondyloarthritis; HLA = human leucocyte antigen; AxSpA = axial spondyloarthritis; PsA = psoriatic arthritis. * Some patients received several different biological therapies before treatment with secukinumab.

**Table 2:** Comparison of inflammatory markers and disease activity at months 0, 3, and 12 of secukinumab treatment in patients with a history of tuberculosis.

	*n*	Month 0	Month 3	Month 12	*p*-Value	0th–3rd Month *p*	3rd–12th Month *p*	0th–12th Month *p*
ESR (mm/h)	17	32 (2–75)	12 (2.1–40)	8 (3–30)	<0.001	<0.001	0.035	<0.001
CRP (mg/L)	17	24 (2.3–79.7)	4.7 (2.2–37)	4 (2–7)	0.003	<0.001	≤0.001	0.155
BASDAI	17	5.6 (5.1–5.9)	3.2 (1.5–5.8)	1.2 (1.1–3.6)	<0.001	<0.001	<0.001	<0.001
ASDAS CRP	17	4.1 (2.4–5.3)	2.4 (1–3.9)	1.2 (1.1–2)	<0.001	<0.001	<0.001	<0.001
VAS (0–10)	17	8 (5–10)	6 (3–8)	3 (2–8)	<0.001	<0.001	<0.001	<0.001

ESR = erythrocyte sedimentation rate, CRP = C-reactive protein, BASDAI = Bath Ankylosing Spondylitis Disease Activity Index, ASDAS = Ankylosing Spondylitis Disease Activity Score, VAS = visual analog scale.

**Table 3:** Characteristics of patients with a history of MS at the start of secukinumab treatment (*n* = 9).

Diagnosis age of SpA, years	37.9 ± 9.6
Male sex, *n* (%)	4 (44.4)
SpA characteristics, *n* (%)	
HLA B27	3 (33.3)
AxSpA	5 (55.6)
PsA	4 (44.4)
Enthesitis	0 (0)
Dactylitis	2 (22.2)
Uveitis	0 (0)
Smoking, *n* (%)	
Current	3 (33.3)
Former	1 (11.1)
Never	5 (55.6)
Alcohol, *n* (%)	
Current	0 (0)
Former	0 (0)
Never	0 (0)
Comorbidity, *n* (%)	
Hypertension	2 (22.2)
Diabetes mellitus	2 (22.2)
Dyslipidemia	3 (33.3)
Familial Mediterranean fever	1 (11.1)
Biological therapies, *n* (%)	
Bionaive	3 (33.3)
Bioexperienced *	6 (66.7)
Etanercept	2 (22.2)
Adalilumab	1 (11.1)
Certolizumab	3 (33.3)
Infliximab	1 (11.1)
Ustekinumab	2 (22.2)
Age at MS diagnosis, years	43.8 ± 8.3
Time between MS and treatment with secukinumab, years	3 ± 2.7
Secukinumab follow up time, months	67.8 (13.4–78.6)

SpA = spondyloarthritis, HLA = human leucocyte antigen; AxSpA = axial spondyloarthritis, PsA = psoriatic arthritis, MS = multiple sclerosis. * Some patients received several different biological therapies before treatment with secukinumab.

**Table 4:** Comparison of secukinumab treatment inflammatory markers and disease activity at months 0, 3, and 12 in patients with history of MS.

	*n*	Month 0	Month 3	Month 12	*p*-Value	0th–3rd Month *p*	3rd–12th Month *p*	0th–12th Month *p*
ESR (mm/h)	9	24 (18–64)	22 (10–30)	8 (4–24)	<0.001	0.012	0.008	0.008
CRP (mg/L)	9	14 (2–40)	6 (2–16.8)	3 (2–8.7)	0.003	0.017	0.028	0.012
BASDAI	7	5.6 (5.3–5.9)	4.2 (3.4–6.5)	2.4 (1.2–5.1)	0.002	0.042	0.018	0.018
ASDAS CRP	7	4 (3.3–4.4)	2.7 (1.5–3.7)	2.2 (1.2–2.8)	≤0.001	0.018	0.018	0.018
VAS (0–10)	9	9 (8–10)	6 (4–7)	3 (0–6)	<0.001	0.007	0.007	0.007

ESR = erythrocyte sedimentation rate, CRP = C-reactive protein; BASDAI = Bath Ankylosing Spondylitis Disease Activity Index, ASDAS = Ankylosing Spondylitis Disease Activity Score, VAS = visual analog scale.

**Table 5:** Characteristics of patients with history of CHF at the start of secukinumab treatment (*n* = 16).

Diagnosis age of SpA, years	40 (33.4–65.9)
Male sex, *n* (%)	12 (75)
SpA characteristics, *n* (%)	
HLA B27	5 (31.3)
AxSpA	13 (81.2)
PsA	3 (18.8)
Enthesitis	4 (25)
Dactylitis	2 (12.5)
Uveitis	1 (6.3)
Smoking, *n* (%)	
Current	6 (37.5)
Former	4 (25)
Never	6 (37.5)
Alcohol, *n* (%)	
Current	3 (18.8)
Former	0 (0)
Never	13 (81.2)
Comorbidity, *n* (%)	
Hypertension	15 (93.8)
Diabetes mellitus	4 (25)
Dyslipidemia	7 (43.8)
Chronic renal failure	7 (43.8)
Coronary artery disease	11 (68.8)
Chronic obstructive pulmonary disease	5 (31.3)
Familial Mediterranean fever	1 (6.3)
Biological therapies, *n* (%)	
Bionaive	9 (56.3)
Bioexperienced *	7 (43.7)
Etanercept	4 (25)
Adalilumab	2 (12.5)
Golilumab	1 (6.3)
Infliximab	2 (12.5)
Age at CHF diagnosis, years	59.1 ± 11
Time between CHF and treatment with secukinumab, years	4.3 ± 3.2
CHF stage, *n* (%)	
NYHA classification	
Stage 2	9 (56.2)
Stage 3	7 (43.8)
LEVF classification	
HFmrEF	11 (68.8)
HFrEF	5 (31.2)
Secukinumab follow up time, months	31.3 (14.3–69.7)

SpA = spondyloarthritis, HLA = human leucocyte antigen, AxSpA = axial spondyloarthritis, PsA = psoriatic arthritis, CHF = congestive heart failure, NYHA = New York Heart Association, LVEF = left ventricular ejection fraction, HFrEF = heart failure with reduced ejection fraction, HFmrEF = heart failure mildly reduced ejection fraction. * Some patients received several different biological therapies before treatment with secukinumab.

**Table 6:** Comparison of secukinumab treatment inflammatory markers and disease activity at months 0, 3, and 12 in patients with history of CHF.

	*n*	Month 0	Month 3	Month 12	*p*-Value	0th–3rd Month *p*	3rd–12th Month *p*	0th–12th Month *p*
ESR (mm/h)	16	28 (9–63)	18 (3.5–77)	12 (4–41)	≤0.001	0.012	0.432	≤0.001
CRP (mg/L)	16	16 (1.8–68)	6.5 (1.7–70)	4 (2–8.1)	<0.001	≤0.001	0.028	≤0.001
BASDAI	15	5.4 (5.2–6.1)	4.2 (2.4–8.6)	1.2 (1.1–3.6)	<0.001	0.140	≤0.001	≤0.001
ASDAS CRP	15	4.6 (3.3–5.2)	3.4 (2–4.5)	1.2 (1.1–2)	<0.001	≤0.001	≤0.001	≤0.001
VAS (0–10)	16	9 (6–10)	6 (2–9)	3 (2–8)	<0.001	≤0.001	≤0.001	<0.001

ESR = erythrocyte sedimentation rate, CRP = C-reactive protein; BASDAI = Bath Ankylosing Spondylitis Disease Activity Index, ASDAS = Ankylosing Spondylitis Disease Activity Score, VAS = visual analog scale.

## Data Availability

The data presented in this study are available on request from the corresponding author.

## Abbreviations

The following abbreviations are used in this manuscript:

AxSpA	Axial spondyloarthritis
PsA	Psoriatic arthritis
SpA	Spondyloarthritis
NSAIDs	Non-steroidal anti-inflammatory drugs
csDMARDs	Conventional synthetic disease-modifying antirheumatic drugs
TNF	Tumor necrosis factor
IL	Interleukin
MS	Multiple sclerosis
CHF	Congestive heart failure
ASAS	Assessment of SpondyloArthritis International Society
CASPAR	Classification criteria for psoriatic arthritis
MR	Magnetic resonance
NYHA	New York Heart Association
LVEF	Left vetnricular ejection fraction
HFrEF	Heart Failure with reduced ejection fraction
HFmrEF	Heart Failure mildly reduced ejection fraction
HLA	Human leukocyte antigen
ESR	Erythrocyte sedimentation rate
CRP	C-reactive protein
VAS	Visual analog scale
BASDAI	Bath Ankylosing Spondylitis Disease Activity Index
ASDAS	Ankylosing Spondylitis Disease Activity Score
DAS 28	Disease Activity Score-28
PASI	Psoriasis Area and Severity Index
